# Antiseptic Treatment in Obstetrics

**Published:** 1887-01

**Authors:** 


Antiseptic Treatment in Obstetrics.—The following is a brief summary of the rules enforced in Prof. Spaeth's Obstetric Clinic in Vienna, and contains many valuable hints that may be followed in general private practice: "Before any vaginal examination, hands must be cleansed with soap and brush, and dipped in from one to five per cent. carbolic. Examining finger smeared with three per cent. carbolic vaseline. Neither before or after examination is vaginal douche given, unless there be special ground therefor—from a bad discharge, etc. After a normal, spontaneous labor, the external genitals are washed with a one to two per cent. carbolic solution. After intra-uterine manipulation, the uterus is washed out with one to two litres warm, one to two per cent. carbolic. After dead fœtus (decomposed) or difficult instrumentation, an iodoform pencil, in addition, is used. Episiotomy wounds, if not deep enough for suture, are simply dusted with iodoform, and similarly slight perineal ruptures. During puerperium, unless especially ordered, neither vaginal injections nor antiseptic compresses. As soon as a lying-in woman has elevation of temperature, she is isolated. Each ward is carefully disinfected when empty. In case of rise of temperature, if there are wounds of the outer genitals, vaginal injections, one to two per cent. carbolic; if lochia are bad-smelling, intra-uterine douche twice daily, or else iodoform pencils. Douche is stopped as soon as lochia become normal. Continuous irrigation never used. Iodoform freely on all wounded surfaces.—*Am. Journal Obstetrics.*

